# Ascorbic acid deficiency promotes metabolic remodeling and pulmonary fibrosis that leads to respiratory failure in Sod1 and Akr1a double-knockout mice

**DOI:** 10.1016/j.redox.2025.103749

**Published:** 2025-07-01

**Authors:** Tsukasa Osaki, Takujiro Homma, Yuya Soma, Satoshi Miyata, Yumi Matsuda, Junichi Fujii

**Affiliations:** a Department of Biochemistry and Molecular Biology, Graduate School of Medical Science, Yamagata University, 2-2-2 Iidanishi, Yamagata, 990-9585, Japan; b Graduate School of Nursing, Yamagata University Faculty of Medicine, 2-2-2 Iidanishi, Yamagata, 990-9585, Japan; c Miyata Diabetes and Metabolism Clinic, 5-17-21 Fukushima, Fukushima-ku, Osaka, 553-0003, Japan

**Keywords:** Superoxide, Pneumonia, Proteomics, Tricarboxylic acid cycle, Urea cycle

## Abstract

We recently reported that mice with a double knockout (DKO) of Sod1 encoding superoxide dismutase 1 (SOD1) and Akr1a encoding aldehyde reductase survived more than one year when supplemented with ascorbic acid (Asc) (1.5 mg/ml in drinking water), and that the withdrawal of Asc resulted in premature death in only two weeks due to oxidative damage-associated pneumonia. SOD1 is known to disable the radical electrons of superoxide, which suppresses the subsequent formation of highly reactive oxygen species (ROS). Akr1a encodes aldehyde reductase, which catalyzes the biosynthesis of Asc, which is a strong nutritional antioxidant. In this study, we sought to gain insight into the metabolic basis for the progression of respiratory failure in the DKO mice. Pathological examinations have revealed pulmonary damage and the progression of fibrosis caused by an elevation in pulmonary cell death in these mice. Metabolite analyses have shown that substrate compounds catabolized in the tricarboxylic acid cycle are shifted from carbohydrates to amino acids, which leads to polyamine synthesis. While proteins involved in cell polarization, adhesion, and transport are increased in the lungs, showing trends similar to those of activated leukocytes, antioxidative enzymes were characteristically decreased in the lungs. Carbonyl proteins were originally high in the DKO mice but did not increase following Asc withdrawal, which was likely caused by stimulation of the degradation of oxidized proteins through the ubiquitin-proteasome system. It is conceivable that the oxidative insult due to Asc insufficiency under Sod1 deficiency causes protein oxidation followed by degradation, which fuels the tricarboxylic acid cycle. Remodeling the metabolic pathways for amino acid use increases polyamine synthesis, which could stimulate pulmonary fibrosis and lead to respiratory failure.

## Highlights

• Ascorbic acid deficiency leads to pulmonary fibrosis in Sod1-and Akr1a-double knockout mice.
• Oxidative insult leads to metabolic remodeling of the TCA cycle in pulmonary tissues.
• Substrates catabolized in the TCA cycle shift from carbohydrates to amino acids.
• Amino acid catabolism leads to polyamine synthesis, which stimulates fibrotic proliferation.

## Graphical abstract

**Figure:**
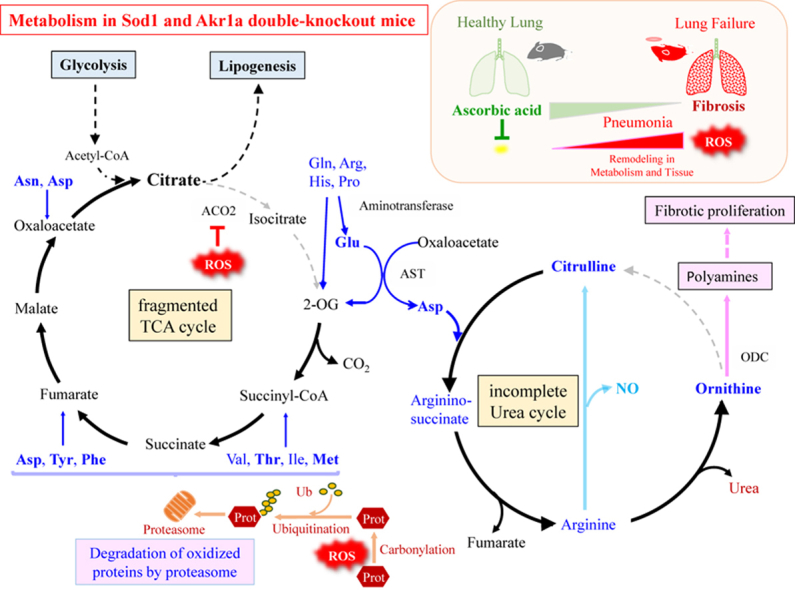

## Abbreviations

ACO1: cytosolic aconitase
ACO2: mitochondrial aconitase
AKR1A: aldehyde reductase
Akr1a: aldehyde reductase gene
Arg: arginine
ARDS: Acute respiratory distress syndrome
Asc: ascorbic acid
Asp: aspartate
AST: aspartate aminotransferase
COPD: chronic obstructive pulmonary disease
DKO: Sod1-and Akr1a-double knockout
EDTA: ethylenediaminetetraacetic acid
ER: endoplasmic reticulum
NEM: *N*-ethylmaleimide
Fe–S: iron-sulfur
Gln: glutamine
Glu: glutamate
GO: gene ontology
H&E: hematoxylin and eosin
HRP: horseradish peroxidase
IL-1β: interleukin-1β
IFNγ: interferon γ
KO: knockout
LPS: lipopolysaccharide
nLC-MS/MS: nanoflow liquid chromatography tandem mass spectrometry
NO: nitric oxide
ODC: ornithine decarboxylase
2-OG: 2-oxoglutarate
PBS: phosphate-buffered saline
PVDF: polyvinylidene difluoride
ROS: reactive oxygen species
SOD1: superoxide dismutase 1
Sod1: superoxide dismutase 1 gene
SE: standard error
TBST: tris-buffered saline containing 0.1 % Tween-20
TCA: tricarboxylic acid
TNF: tumor necrosis factor
WBC: white blood cells
WT: wild type

## 1 Introduction

Lungs are continuously exposed to the highest oxygen concentration in the body, and, hence, are considered to be the first organ that suffers oxidative damage under systemic dysfunction of the antioxidative system [1]. Accordingly, oxidative stress is reportedly associated with a variety of pulmonary diseases [2]. Superoxide dismutase (SOD) is a primary antioxidative enzyme that suppresses the secondary generation of other reactive oxygen species (ROS) in cooperation with peroxidases such as catalase and glutathione peroxidase [3]. Among three known SOD isozymes, SOD1 is localized in the cytosol and in mitochondrial intermembrane spaces. The phenotypic abnormalities of Sod1-knockout (KO) mice, however, have proven to be less severe than initial expectations [4]. Representative phenotypic abnormalities are enhanced by the death of neuronal cells following axonal injury [5], female infertility [6], and an increased incidence of liver tumors manifested by a shorter lifespan [7]. In contrast, in *ex vivo* situations a Sod1 deficiency is marked by abnormalities in certain types of cells. For instance, fibroblasts derived from Sod1-KO mice undergo apoptotic death [8,9], and Sod1-KO embryos are developmentally arrested at the two-cell stage under cultured conditions [10]. These contradictory observations between *in vivo* and *ex vivo* situations are considered to be partially attributable to the humoral antioxidant system in the body, which may include enzymes such as extracellular SOD encoded by Sod3 and extracellular glutathione peroxidase encoded by Gpx3 and antioxidant compounds such as bilirubin, uric acid, and antioxidant vitamins [3,4,9].

Ascorbic acid (Asc), also called vitamin C in primates, is present in large amounts (∼1 mM) in organelles such as the endoplasmic reticulum (ER) and mitochondria [11,12] where it exhibits strong levels of antioxidant activity [13,14]. Asc preferentially reacts with radical species and is converted into ascorbyl radical. Ascorbyl radical is less reactive to many biological compounds, due to the resonance stabilization, but still eliminates radicals by donating an electron to other oxygen radicals, resulting in dehydroascorbate [13,14]. Humans and other primates cannot synthesize Asc, so they must ingest it through their diet, for example through fruits and vegetables. Administration of antioxidants including Asc is reportedly an effective treatment for patients with injurious situations of the lungs [15]. For instance, acute respiratory distress syndrome (ARDS), which is a devastating clinical condition with a high rate of mortality (30–50 %) and developed in conditions such as sepsis and COVID-19, appears to be effectively treated with Asc [[16], [17], [18]]. However, there remains ambiguity concerning the mechanism of the therapeutic effects of Asc, and verification employing model animals is anxiously awaited.

Primates cannot synthesize Asc due to a mutation of Gulo, the gene encoding L-gulono-γ-lactone oxidase, whereas rodents have retained the ability to synthesize Asc [19]. Aldehyde reductase encoded by Akr1a catalyzes the reductive conversion of d-glucuronic acid to l-gulonate in the Asc-synthesizing pathway, which contributes to 85–90 % of the Asc synthesis in mice [20,21], and another isoform aldose reductase encoded by Akr1b contributes the remaining 10–15 %. Accordingly, mice with an Akr1a knockout (Akr1a-KO) produce approximately 10 % of the Asc that is produced in wild-type (WT) mice, which is insufficient to meet the physiological requirements of Asc [22]. As a result, Akr1a-KO mice show aberrant phenotypes such as defects in osteogenesis due to impaired collagen synthesis [20] and a lifespan shortened to less than one year [23]. These defects are related mostly to Asc insufficiency.

We hypothesized that the presence of abundant levels of Asc contributes to the elimination of ROS and mitigation of oxidative damage in Sod1-KO mice and, to verify this notion, established double knockout (DKO) mice that lack both Sod1 and Akr1a [24]. DKO mice could survive longer than one year via the use of Asc supplementation, but, following Asc withdrawal, they undergo premature death within two weeks due to acute pneumonia, regardless of age and gender [24]. The results clearly indicate that endogenously produced Asc actually obscures the fatal damage caused by the ROS that are elevated in a Sod1-deficient background. This notion is also supported by findings showing that the administration of Asc reverses the lethality of the primary fibroblasts in Sod1-KO mice [9] and restores the developmental competence of cultured Sod1-KO embryos [25]. However, the metabolic background by which elevated ROS lead to respiratory failure remains unclear. In this study, we intended to gain insight into the metabolic basis for the progression of acute pneumonia in DKO mice following Asc withdrawal. We believe that the results of this study will help elucidate the mechanism of death in DKO mice, and also will provide clues to understanding the metabolic background involved in the progression of pulmonary diseases caused by oxidative stress.

## 2 Materials and methods

### 2.1 Animals

All of the mice with a C57BL/6N background were weaned at 30 days of age and fed a standard diet (LabDiet 5053; PicoLab, St Louis, MO, USA) *ad libitum*. DKO mice lacking both Sod1 [26] and Akr1a [21] were generated by mating Sod1-KO mice with Akr1A-KO mice and were bred by administering Asc (1.5 mg/ml) in their drinking water, as described previously [24]. For treatment with the proteasome inhibitor MG132 (ChemScene, NJ, USA), mice were administered 2 mg/kg MG132 intraperitoneally once every two days.

The animal room was maintained under specific pathogen-free conditions at a constant temperature of 20–22 °C with a 12-h alternating light-dark cycle. All mice were bred in the Animal Center, Institute for Promotion of Medical Science Research, Yamagata University Faculty of Medicine. Animal experiments were performed in accordance with the Declaration of Helsinki under the protocol approved by the Animal Research Committee at our institution.

### 2.2 Histopathological evaluation of lung-tissue damage

The lungs were harvested after sacrifice, immersed immediately in a 10 % formalin solution diluted with phosphate-buffered saline (PBS), and fixed for 3 days at room temperature. They were then stored at room temperature in a 70 % ethanol solution until paraffin embedding. Lung sections (5 μm in thickness) were subjected to staining with hematoxylin and eosin (H&E) or Elastica-Masson to evaluate the damage to lungs. TUNEL staining was performed on lung sections using an MEBSTAIN Apoptosis TUNEL kit direct (MBL, Sunnyvale, CA, USA) according to the manufacturer's instructions and counterstained with methyl green. For immunohistochemical analysis, lung sections were incubated with 3 % H_2_O_2_ for 10 min to block endogenous peroxidase activity, followed by antigen retrieval. Antigen retrieval in citrate buffer (pH 6) was performed at 100 °C for 40 min, 60 °C for 40 min, and room temperature for 20 min. After blocking with 5 % skim milk for 2 h at room temperature, sections were incubated overnight at 4 °C with primary rabbit polyclonal antibodies diluted in 1 % bovine serum albumin. Anti-α-smooth muscle actin (SMA) antibody (14395-1-AP, Proteintech Japan, Tokyo, Japan) was used at a dilution of 1:400, anti-myeloperoxidase (MPO) antibody (22225-1-AP, Proteintech Japan) was used at a dilution of 1:3200, anti-nitric oxide synthase 2 (NOS2) antibody (22226-1-AP, Proteintech Japan) was used at a dilution of 1:800, anti-CD80 antibody (bs-1479R, Bioss Inc., Woburn, MA, USA) was used at a dilution of 1:1600, and anti-CD163 antibody (bs-2527R, Bioss Inc.) was used at a dilution of 1:800. Sections were incubated with Histofine Simple Stain Mouse Max PO (R) (Nichirei Bioscience, Tokyo, Japan) for 1 h and then stained with a color developing reagent (DAB Solution, Nichirei Bioscience) for 10 min at room temperature. Nuclear staining was performed by incubation with hematoxylin for 10 min at room temperature. Images were obtained with a BZ-X700 fluorescence microscope (KEYENCE, Osaka, Japan), and nuclei stained dark brown were quantified using ImageJ software (http://imagej.nih.gov/ij/) [27].

### 2.3 Blood cell counting

Blood was collected from the tail veins in the presence of ethylenediaminetetraacetic acid (EDTA). Hematological examination was carried out using an automated blood cell analyzer (VetScan HM5 v2.31, Abaxis, CA, USA). Red blood cells, platelets, and total white blood cells (WBCs) were separately counted, and WBCs were further classified as either lymphocytes, monocytes, or granulocytes.

### 2.4 Collecting plasma and preparation of lung-tissue lysate

Blood collected in the presence of EDTA was centrifuged at 2400×*g* for 5 min, and the plasma fraction was then subjected to analysis. Lung tissues dissected from mice were homogenized in a lysis buffer (25 mM Tris-HCl, pH 7.5, containing 150 mM NaCl, 1 % NP-40, 1 % sodium deoxycholate, and 0.1 % SDS supplemented with a protease inhibitor cocktail (P8340; Sigma-Aldrich, St. Louis, MO, USA)) using a glass-Teflon homogenizer on ice with centrifugation at 17,400×*g* under a temperature of 4 °C. Protein concentrations of the supernatant were determined using a Pierce® BCA™ protein assay kit (Thermo Fisher Scientific, Waltham, MA, USA).

### 2.5 Measurement of interleukin-1β (IL-1β) and interferon γ (IFNγ) in blood plasma and lung tissue

IL-1β and IFNγ in blood plasma, lung lysate, and macrophage-cultured media were measured via a BD™ Cytometric Bead Array Flex Set (BD Biosciences, San Jose, CA, USA) using FACSCanto II (BD Biosciences), as described previously [24]. Data on the lung tissues were expressed per protein content of the samples.

### 2.6 Isolation of macrophages from mice followed by treatment with lipopolysaccharide (LPS)

Peritoneal macrophages were collected and cultured as previously described [28]. Briefly, mice were injected intraperitoneally with 2 mL of 4 % thioglycolate broth. Four days after the injections, macrophages were collected by peritoneal lavage. After being counted, the cells were plated at 2 × 10^6^ cells/12-well plates in complete RPMI 1640 medium containing 10 % fetal bovine serum, 100 units/mL penicillin, and 100 units/mL streptomycin. After 2 h, the medium was replaced to remove non-adherent cells, and the remaining cells were cultured for 24 h and stimulated with LPS (1 μg/mL; 125–05181 Fujifilm Wako Pure Chemical Corp). After 24 h, all culture medium was collected and the cells were harvested with Cell Dissociation Solution (Biological Industries, Cromwell, CT, USA) according to the manufacturer's instructions.

### 2.7 Measurement of nitrite in cultured media

The levels of nitrites, oxidized metabolites derived from nitric oxide (NO), in cultured media were assessed by means of the Griess reaction, as previously described [28]. The nitrite content in the samples was determined using NaNO_2_ as the standard.

### 2.8 Metabolite analysis

Sample preparation and metabolite measurements were performed as described [29] with minor modifications. Briefly, alkylation was performed by treatment with 20 mM N-ethylmaleimide (NEM) in 50 mM ammonium bicarbonate. Equal volumes of methanol containing both 10 μM of NEM-derivatized glutathione and 10 μM of l-methionine sulfone were added as internal standards. Following the addition of an equal volume of chloroform, the mixtures were centrifuged at 12,000×*g* for 15 min at 4 °C. The upper aqueous layer was lyophilized, dissolved in a one-third volume of deionized water, and analyzed via nanoflow liquid chromatography tandem mass spectrometry (nLC-MS/MS). A Q-exactive Hybrid Quadrupole-Orbitrap mass spectrometer (Thermo Fisher Scientific) equipped with a heated electrospray ionization source was operated in both the positive and negative ionization modes. The Ultimate 3000 LC system consisted of a WPS-3000 TRS autosampler, a TCC-3000 RS column oven, and an HPG-3400RS quaternary pump (Dionex, Sunnyvale, CA, USA). A SeQuant ZIC-pHILIC column (2.1 × 150 mm, 5 μm particle size; Merck KGaA, Darmstadt, Germany) and an Acquity UPLC BEH Amide column (2.1 × 100 mm, 1.7 μm particle size; Waters Corp., Milford, MA, USA) were used to quantify as many metabolites as possible. For the ZIC-pHILIC column, mobile phase A consisted of 20 mM ammonium bicarbonate, pH 9.8, and mobile phase B was 100 % acetonitrile. For the BEH Amide column, mobile phase A consisted of 0.1 % formic acid and mobile phase B was 99.9 % acetonitrile containing 0.1 % formic acid. System control and data acquisition were performed using Xcalibur 2.2 software.

All raw data were collected and imported into Compound Discoverer 2.1 software (Thermo Fisher Scientific) for compositional determination. Elemental compositions were searched using Compound Discoverer 2.1 against the mzVault metabolite database, which was built in February 2017 based on accurate mass and isotopic patterns. Tentative metabolite identification was performed by comparing the observed full MS ions and MS/MS fragment ions, and validated identification was performed using reference standards. Compounds were grouped with a mass tolerance of 20 ppm and a retention-time tolerance of 1 min and were quantified based on the relative normalized peak area of each signal in the mass spectrum.

### 2.9 Proteomics analyses

Lung lysate samples (50 μg) were reduced using dithiothreitol (10 mM) followed by alkylation with iodoacetamide (25 mM). Following hydrolysis with trypsin, reaction mixtures were desalted using a C-Tip (Nikkyo Technos, Tokyo, Japan), as previously described [30]. The desalted peptide solutions were analyzed via nLC-MS/MS using the Easy nLC 1000 system (Thermo Fisher Scientific) connected to a quadrupole orbitrap mass spectrometer (Q-Exactive; Thermo Fisher Scientific) equipped with a nanoelectrospray emitter. The measurement conditions were previously described [31].

Raw file reads were matched against the Swiss-Prot house mouse database (17,162 sequences), using Proteome Discoverer (version 1.4; Thermo Fisher Scientific) with the Sequest^HT^ and Mascot (version 2.8.0.1; Matrix Science, Tokyo, Japan) search engines. Precursor and fragment mass tolerances were set to 10 ppm and 0.04 Da, respectively. Fixed modification for S-carbamidomethylated cysteine and two maximum missed cleavage sites for trypsin were set. For identification of oxidized modification, variable modification for carbonylated arginine; carbonylated lysine; carbonylated proline and/or conversion of proline to pyroglutamic acid; cysteine sulfenic acid, cysteine sulfonic acid, and/or *S*-nitrosyl cysteine; or methionine sulfoxide and/or methionine sulfone were set. The results were filtered using a Percolator with a false discovery rate of 1 %. The peak area of each identified peptide was estimated using Proteome Discoverer. The intensity of unique peptides was used to calculate the protein intensity. An intensity-based absolute quantification (iBAQ) algorithm was used to calculate the protein quantification values [32].

### 2.10 Protein data annotation

For gene ontology (GO) analysis, we used two approaches to select “upregulated” proteins (34 proteins, bold red in Supplementary Table 1). First, we selected proteins upregulated in DKO (day 7) *vs.* WT, but not in DKO (day 0) *vs.* WT (10 + 5 proteins; Fig. 7B, left, bold). We also selected proteins upregulated in DKO (day 10) *vs.* WT, but not in DKO (day 0) *vs.* WT (19 + 5 proteins; Fig. 7B, left, bold). There are five proteins common to the two groups, so the total is a net 34 proteins. The criteria for an “upregulated” protein is a ratio greater than 2-fold and a *P*-value less than 0.05. For selection of “downregulated” proteins (15 proteins, bold blue in Supplementary Table 1), we also used two approaches. We selected proteins downregulated in DKO (day 7) *vs.* WT, but not in DKO (day 0) *vs.* WT (6 + 6 proteins; Fig. 7B, right, bold). We also selected proteins downregulated in DKO (day 10) *vs.* WT, but not in DKO (day 0) vs. WT (3 + 6 proteins; Fig. 7B, right, bold). There are six proteins common to the two groups, so the total is a net 15 proteins. The criteria for a downregulated protein is a ratio less than 0.5-fold and a *P*-value less than 0.05. Gene ontology (GO) analyses of the differentially expressed genes were performed using protein analysis *via* the evolutionary relationships (PANTHER) classification system (GO database Released 2025-02-06). *P*-values of each GO term above 0.05 were excluded from the analysis. The number of differentially expressed genes for particular GO terms was compared with the total number of genes assigned to each term, and the enriched GO terms were presented. Differentially expressed genes were categorized as “biological processes”, “molecular function”, and “cellular component”.

### 2.11 Detection of carbonylated proteins

Levels of protein carbonyl groups were assessed using a protein carbonyl assay kit (Abcam, Cambridge, MA, USA) according to the manufacturer's instructions. Lung lysate samples (5 mg/mL) were chilled on ice for 20 min with an equal volume of 2 x extraction buffer and centrifuged at 17,400×*g* for 20 min. The supernatant was mixed with an equal volume of 12 % SDS, incubated with 2,4-dinitrophenylhydrazine solution for 15 min at room temperature, and then neutralized. Samples (1 μg) were separated on 12 % SDS-polyacrylamide gel and blotted onto a polyvinylidene difluoride (PVDF) membrane (GE Healthcare, Chicago, IL, USA). Blots were blocked and incubated with anti-DNP antibody, followed by horseradish peroxidase (HRP)-conjugated secondary antibody. After washing with Tris-buffered saline containing 0.1 % Tween-20 (TBST), the antibody binding was visualized using an Immobilon western chemiluminescent HRP substrate (Merck Millipore, Burlington, MA, USA) and detected using an image analyzer (ImageQuant LAS 500; GE Healthcare). Quantification of signal intensity was performed using ImageJ software.

### 2.12 Detection of ubiquitinated proteins

The lung lysate samples (20 μg) were separated on SDS–polyacrylamide gels and blotted onto PVDF membranes. The blots were blocked with 5 % skim milk in TBST, and then incubated overnight at 4 °C with mouse monoclonal antibody raised against ubiquitin (Ub) (sc-8017; Santa Cruz Biotechnology, Dallas, TX, USA) and diluted in TBST containing 5 % skim milk. After a 3 washes with TBST, the blots were incubated with HRP-conjugated anti-mouse (sc-2005; Santa Cruz Biotechnology) secondary antibody. Positive signals were visualized and quantified as described above.

### 2.13 Measurement of free iron in lung homogenate

Free iron concentrations in the lungs were determined by observing visible coloration due to the formation of a chelate complex between ferrozine and iron using an iron assay kit (Metallo assay; Metallogenics Co., Ltd., Chiba, Japan) according to the manufacturer's instructions. Briefly, the lung lysate was adjusted to pH 2–3 by adding hydrochloric acid, centrifuged at 15,000 rpm for 15 min, and the supernatant was collected. Iron concentration in the supernatant was determined by measuring the iron-ferrozine complex at a wavelength of 562 nm. Lung iron concentrations were corrected for total protein content.

### 2.14 Measurement of aconitase activity

Aconitase activity was measured via a coupled enzyme reaction in which citrate is converted to isocitrate using an aconitase activity assay kit (MAK051, Sigma-Aldrich, St. Louis, MO, USA) according to the manufacturer's instructions. Briefly, lung tissues were homogenized in an ice-cold assay buffer using a glass-Teflon homogenizer and centrifuged at 800×*g* for 10 min at 4 °C. Samples were diluted 2-fold in assay buffer, and 50 μL samples were transferred to 96-well microplates. Reaction mixtures containing an enzyme mix and the substrate (50 μL) were mixed with each sample and incubated at 25 °C for 45 min. Developer (10 μL) was added to each well, mixed, and incubated at 25 °C for 10 min. The absorbance at 450 nm was measured and the amounts of isocitrate generated were calculated from a standard curve. The activities were normalized according to total protein content.

### 2.15 Statistical analysis

Statistical analyses were performed using JMP version 12.2.0 software (SAS Institute, Cary, NC). All results are expressed as mean ± standard error (SE) of at least triplicate experiments. Statistical analyses were performed using a Student's t-test for comparisons of two groups or one-way analysis of variance (ANOVA), which was followed by a Tukey-Kramer test for comparisons of multiple groups. *P-*values less than 0.05 were considered significant.

## 3 Results

### 3.1 Induction of alveolar tissue injury and subsequent fibrosis in DKO mice after withdrawal of Asc

**Fig. 1:**
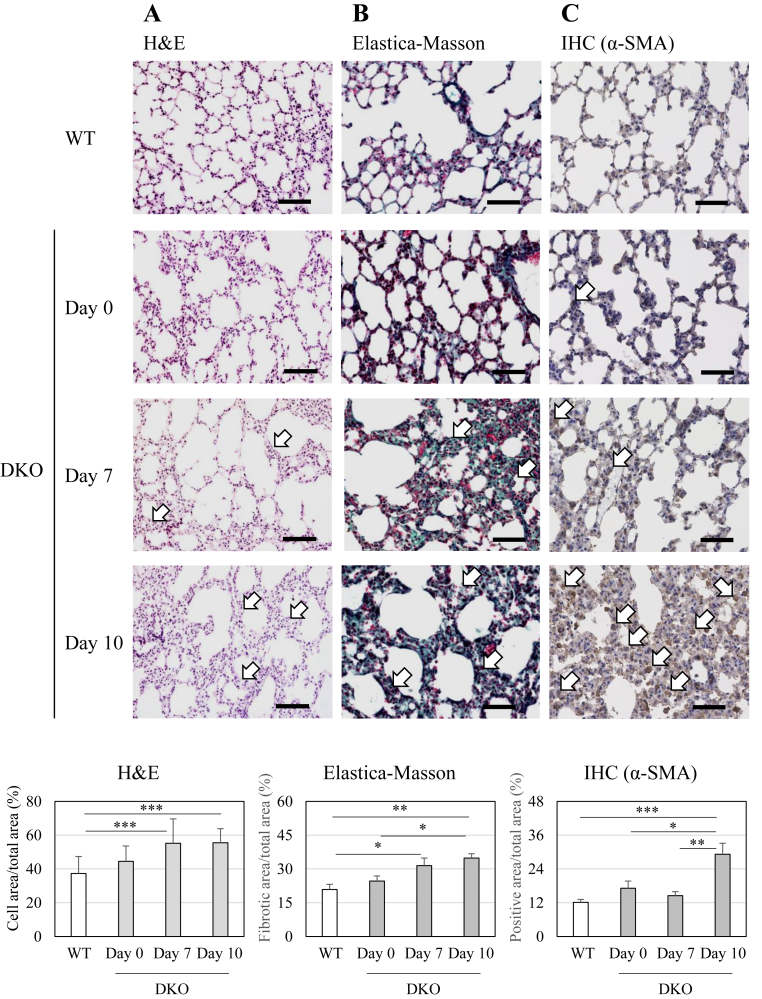
Histological evaluation of the lung tissue of WT mice and DKO mice following Asc withdrawal Mice were euthanized at the indicated time points. Lung sections (5 μm in thickness) were subjected to H&E staining (A), Elastica-Masson staining (B), and immunohistochemical staining with anti-α-smooth muscle actin (α-SMA) antibody (C). The thickened alveolar walls (A), stained collagen fibers (B), and α-SMA-positive areas (C) are indicated by open arrows. The number of animals is 3 each. bar: 100 μm.

**Fig. 2:**
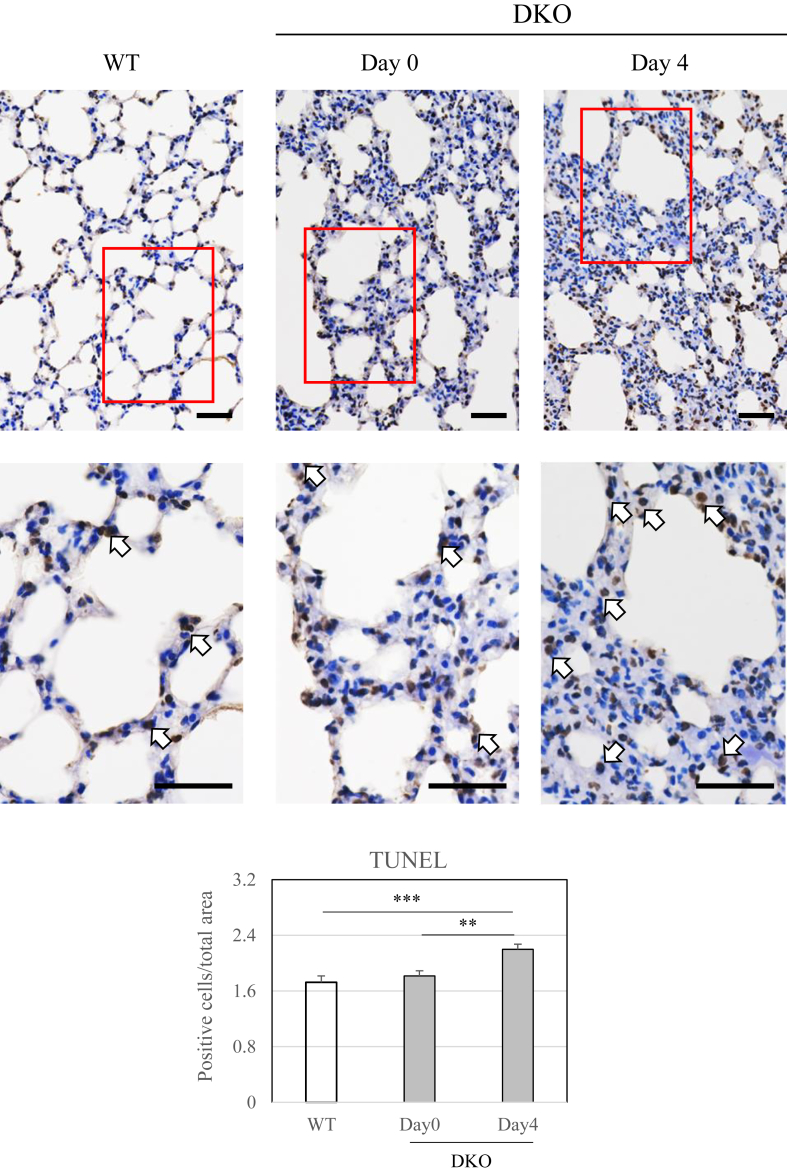
TUNEL staining of the lung tissue of WT mice and DKO mice following Asc withdrawal Top panels are representative images of TUNEL-stained lungs from WT and DKO mice. Cells with dark brown-stained nuclei were considered TUNEL positive, as indicated by arrows. The bottom panels show magnified images of the area outlined in red above. Numbers of positive nuclei were quantified using ImageJ software and expressed per cell area. ∗∗, *P* < 0.01; ∗∗∗, *P* < 0.001. bar: 50 μm.

Because the DKO mice cannot survive beyond two weeks without Asc supplementation [24], they were bred by administering Asc (1.5 mg/ml) in drinking water. All experiments were performed using the female mice, as female DKO mice show a higher survival rate than male DKO mice supplemented with Asc. Following Asc withdrawal at 3–4 months of age, body weight and food intake were decreased in a few days, and all DKO mice eventually died with acute pneumonia after two weeks. We performed histological analyses at several time points to see the progression of pulmonary tissue damage in the DKO mice following Asc withdrawal. Although the lungs of the Asc-supplemented DKO mice (day 0) showed normal appearance, H&E staining of the lung sections revealed that extensive alveolar damage had already begun by day 7, and showed aggravation by day 10 (Fig. 1A). Elastica-Masson staining revealed fibrotic change, which was revealed by blue-green coloration in alveolar tissues following Asc withdrawal (Fig. 1B). We next performed immunostaining using an antibody against α-SMA, a marker protein of myofibroblasts involved in fibrosis, and found that the α-SMA-positive cells gradually increased after Asc withdrawal (Fig. 1C). TUNEL staining was performed on the lung tissues, which shows increases in number of dead cells on day 4 after Asc withdrawal (Fig. 2). Thus, Asc insufficiency under Sod1 deficiency appeared to cause respiratory failure by alveolar cell damage followed by fibrosis.

### 3.2 Asc withdrawal transiently decreased blood lymphocytes but did not increase the proinflammatory cytokines IL-1β and IFNγ in DKO mice

Since mice with a Sod1-deficiency are anemic [26] and WBCs play pivotal roles in the pathogenesis of pneumonia [33], we evaluated the abundance of blood cells in the DKO mice on days 0, 4, 7, and 10 following Asc withdrawal and compared these with those of the WT mice. The DKO mice originally showed low red blood cell concentrations (Supplementary Fig. 1), which are phenotypic abnormalities characteristically observed in mice with a Sod1 deficiency [26]. In the meantime, concentrations of WBCs, particularly the lymphocytes that account for the majority in mice, were higher in the DKO mice even under supplementation with Asc (day 0) compared with those in the WT mice. Following Asc withdrawal, concentrations of lymphocytes were transiently decreased on day 4, which appeared to be a consequence of either infiltration to tissues with inflammation or premature death, but were restored on day 10 probably through hematopoiesis.

**Fig. 3:**
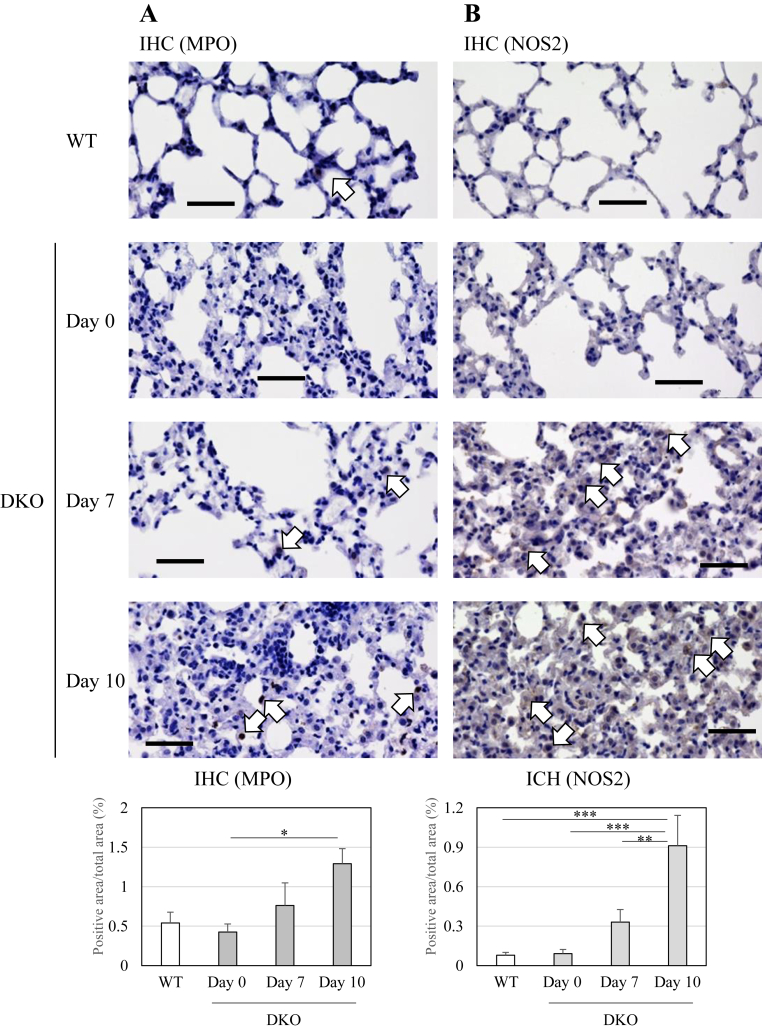
Immunohistochemical staining of the lung tissue of WT mice and DKO mice following Asc withdrawal Lung sections (5 μm in thickness) were subjected to immunohistochemical staining with anti-myeloperoxidase (MPO) antibody (A) and anti-nitric oxide synthase 2 (NOS2) antibody (B). The MPO-positive areas (A) and NOS2-positive areas (B) are indicated by open arrows, respectively. The number of animals is 3 each. bar: 50 μm.

We also performed immunostaining using antibodies against the neutrophil marker protein MPO, the pro-inflammatory macrophage marker proteins NOS2 and CD80, and the anti-inflammatory macrophage marker protein CD163. MPO- and NOS2-positive cells gradually increased after Asc withdrawal (Fig. 3). Although the positive rates for CD80-positive cells and CD163-positive cells were low, a similar tendency was observed (Supplementary Fig. 2). These results indicate that neutrophils and macrophages circulating in the blood infiltrate the lungs after Asc withdrawal.

**Fig. 4:**
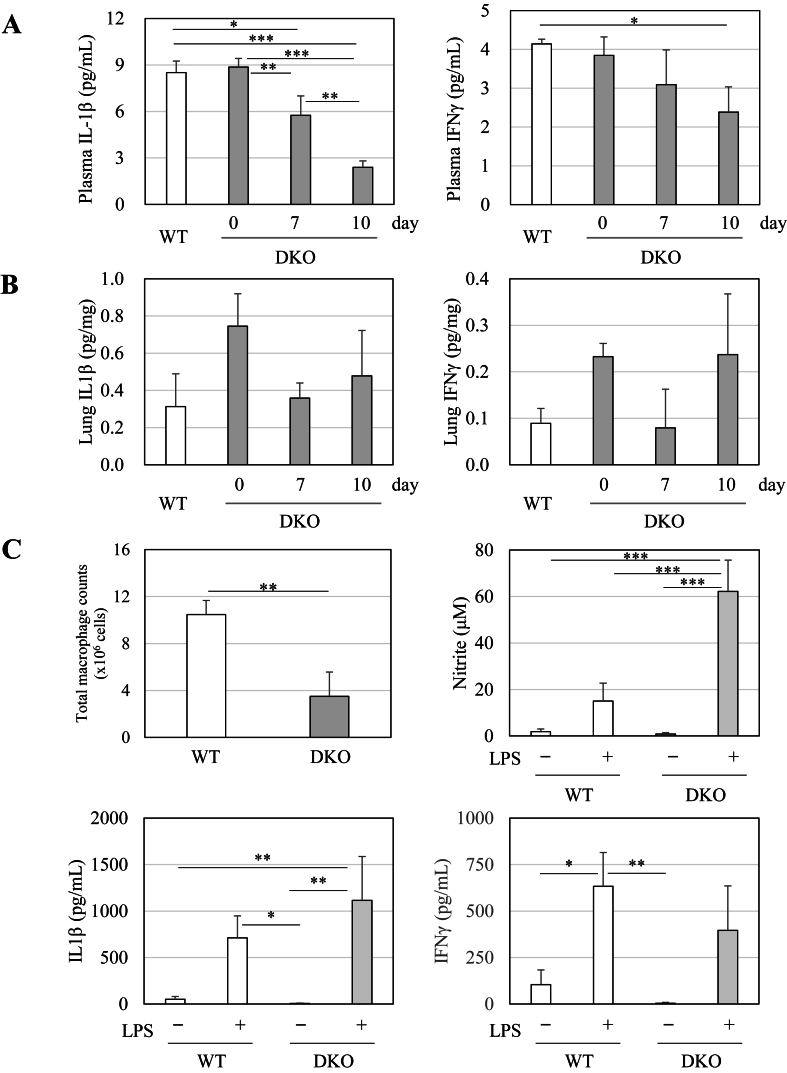
Changes in IL-1β and IFNγ in the blood and lungs of WT and DKO mice following Asc withdrawal IL-1β and IFNγ in blood plasma (A), lung extracts (B), and cultured media of primary macrophages with or without LPS treatment (C) were measured *via* flow cytometry. (C) Total number of collected macrophages and concentration of nitrite in the cultured media of primary macrophages are shown. The number of animals is 3 each. Data are presented as the mean ± SE. ∗, *P* < 0.05; ∗∗, *P* < 0.01; ∗∗∗, *P* < 0.001.

While activated neutrophils dominantly produce ROS through NADPH oxidase, activated macrophages excessively produce inflammatory cytokines as well as ROS, which could cause alveolar damage [34]. Cytokine storm is often assumed in the aggravation of pulmonary diseases including ARDS [35], so we measured the representative pro-inflammatory cytokines. It was surprising that measurements of IL-1β and IFNγ showed decreases rather than increases (Fig. 4A). Similar trends were also observed in the lung tissues (Fig. 4B).

With stimulation of the macrophages, caspase 1 proteolytically activates IL-1β and IL-18, the latter of which then induces IFNγ and tumor necrosis factor (TNF) [36]. Since proteolytic maturation of IL-1β is reportedly defective in Sod1-deficient macrophages due to oxidative modification of the redox-sensitive cysteine residues of caspase 1 [37], we examined this possibility by employing isolated peritoneal macrophages from DKO mice for comparison with those from WT mice. While the total counts of macrophages collected from the DKO mice were less, following stimulation with LPS for 24 h, the production of NO was much higher, which is judged to be a hallmark of activated macrophages (Fig. 4C). The stimulated NO production appeared to be a consequence of strong oxidative stress [38]. Upon LPS stimulation, the levels of IL-1β and IFNγ in cultured media were markedly elevated, although the levels were not significantly different between WT and DKO macrophages. There was no difference in the amount of monocytes in the blood between them, which suggests the premature death of DKO macrophages upon activation in the mice. Since activated Sod1-KO macrophages are less viable, which has been reported by our experiment using peritoneal macrophages [38], the premature death of activated DKO macrophages due to extreme oxidative stress could have impaired the production of IL-1β and IFNγ in an *in vivo* situation. The large number of infiltrated leukocytes appeared to cause alveolar cell death without significant elevation of these proinflammatory cytokines in the DKO mice.

### 3.3 Asc withdrawal caused metabolic alteration in the tricarboxylic acid (TCA) cycle and promotion of the urea cycle through elevated amino acid catabolism in the lung

**Fig. 5:**
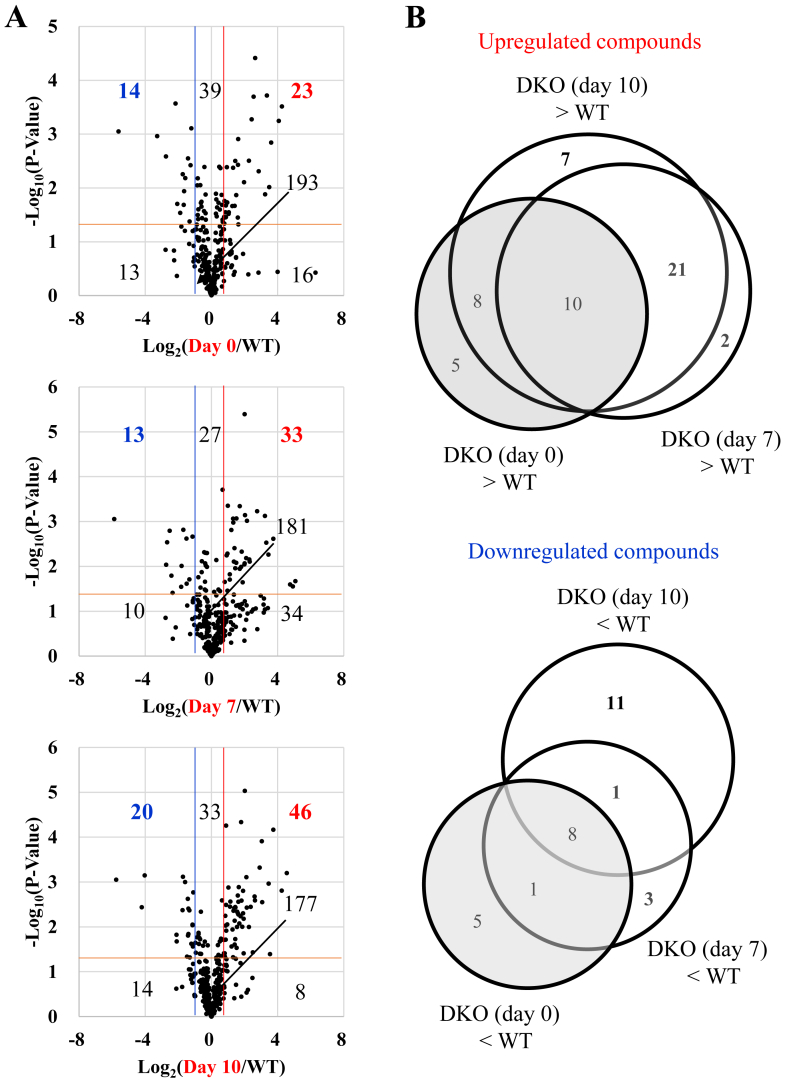
Comparison of low molecular weight compounds with significant changes in metabolite analysis between mice groups Soluble components were extracted from lung tissues and subjected to analyses by means of nLC-MS/MS. (A) Volcano plot of data indicates comparison of metabolites in DKO (day 0) *vs.* WT (top panel), DKO (day 7) *vs.* WT (middle panel), and DKO (day 10) *vs.* WT (bottom panel). The blue and red lines indicate 0.5- and 2-fold levels of DKO (day 0) against WT; of DKO (day 7) against WT; and of DKO (day 10) against WT, respectively. (B) Circular diagram indicates the numbers of metabolites, which were either upregulated or downregulated between two mouse groups. The number of animals is 3 each.

Since ROS largely affect metabolic pathways and could cause their remodeling, as typically seen in macrophages [39], we performed analyses of small metabolic compounds in the tissue homogenates of the mouse lungs. We identified and quantified 293 metabolites (Supplementary Table 2), of which 54 metabolites were verified by comparison with commercially available preparations (Supplementary Table 3). The metabolite volcano plots indicated that 23 metabolites were increased and 14 metabolites were decreased in Asc-supplemented DKO mice (day 0) compared with WT mice (Fig. 5A). In the data for DKO mice (day 7), 33 metabolites were increased and 13 metabolites were decreased compared with WT mice, and in the data for DKO mice (day 10), 46 metabolites were increased and 20 metabolites were decreased compared with WT mice. There were 23 metabolites that were not significantly increased in DKO mice (day 0) but significantly increased in DKO mice (day 7), 28 metabolites that were not significantly increased in DKO mice (day 0) but significantly increased in DKO mice (day 10), and 21 metabolites that were commonly increased (Fig. 5B, bold). On the other hand, there were 4 metabolites that were not significantly decreased in DKO mice (day 0) but significantly decreased in DKO mice (day 7), 12 metabolites that were not significantly decreased in DKO mice (day 0) but significantly decreased in DKO mice (day 10), and 1 metabolite that was commonly decreased. The number of metabolites that increased by Asc withdrawal was greater than the number of the metabolites that decreased.

**Fig. 6:**
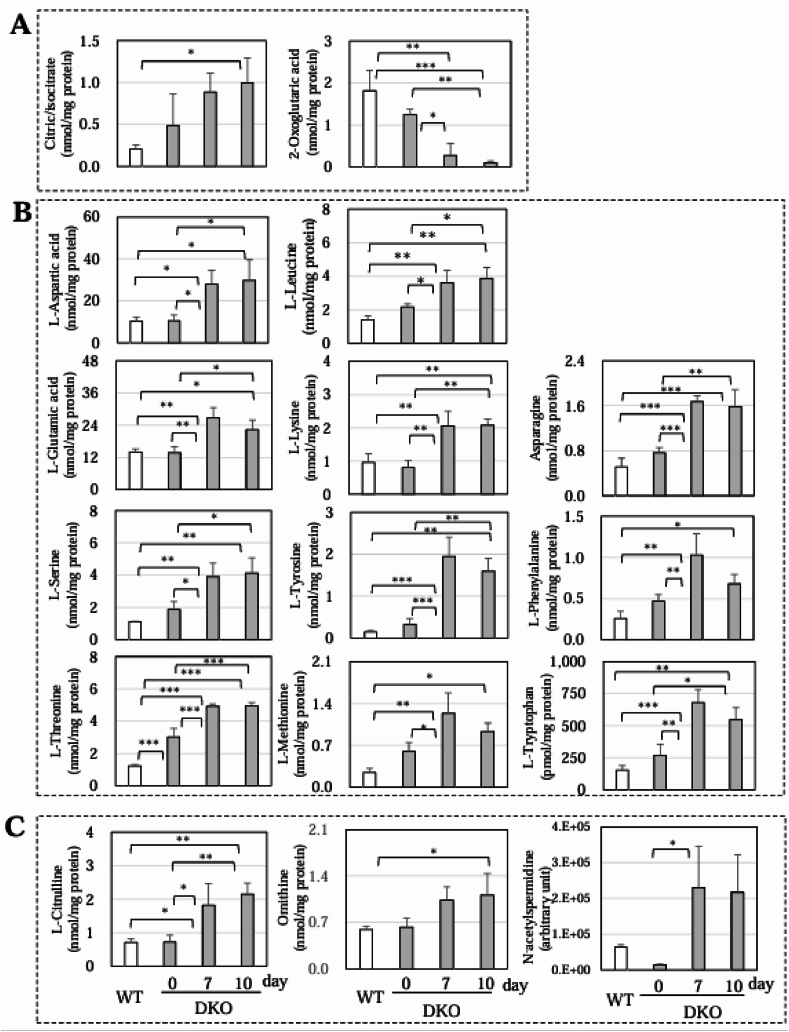
Compounds related to the TCA cycle, amino acids, or the urea cycle among those with significant changes Among the compounds with significant changes in Fig. 5, those related to (A) the TCA cycle, (B) amino acids, and (C) the urea cycle are shown as graphs. Data are presented as the mean ± SE. The number of animals is 3 each. ∗, *P* < 0.05; ∗∗, *P* < 0.01; ∗∗∗, *P* < 0.001.

A heat map of these metabolites revealed evident changes in the compounds, which were associated with the TCA cycle, amino acids, and the urea cycle/polyamine synthesis (Supplementary Fig. 3). Concerning metabolites in the TCA cycle, the citrate/isocitrate content was increased, but that of 2-oxoglutarate (2-OG) was decreased in the DKO mice following Asc withdrawal (Fig. 6A). These results suggest that the TCA cycle was impaired mainly at the stage before 2-OG production. In the meantime, the contents of proteinaceous amino acids were largely upregulated in the DKO mice (Fig. 6B). This could be explained by either enhanced proteolysis or the suppression of amino acid metabolism. The data also show that citrulline and ornithine, amino acids unique to the urea cycle, were elevated in DKO mice following Asc withdrawal (Fig. 6C). It is also noteworthy that the levels of polyamines that could be synthesized from ornithine through the polyamine-synthesizing pathway tended to increase, and a metabolite of polyamine, N^1^/N^8^ acetylspermidine, was markedly elevated. These collective data imply that the substrate utility by the TCA cycle changed from carbohydrates to amino acids and that nitrogen metabolism by polyamine synthesis coupled with the urea cycle was stimulated in the DKO mice following Asc withdrawal.

**Fig. 7:**
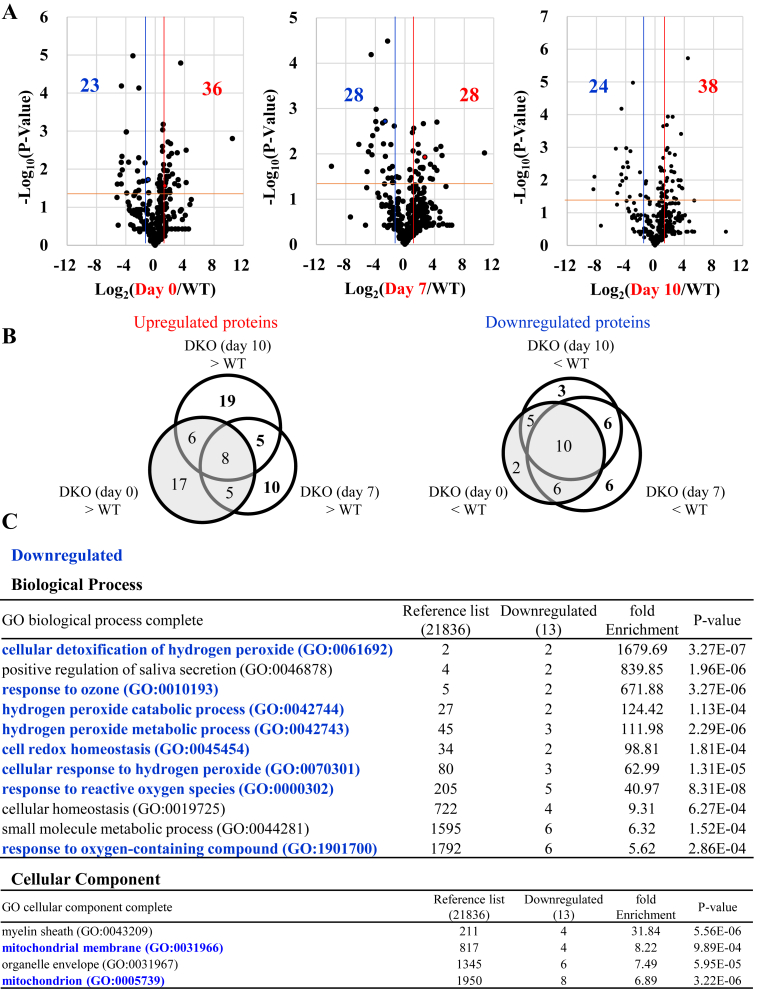
Comparison of proteins with significant changes between mice groups Proteins were extracted from lung tissues and subjected to trypsinization followed by proteomic analyses by means of nLC-MS/MS. (A) The volcano plot of the data indicates the comparison of proteins in WT vs. DKO at day 0 (left), WT vs. DKO at day 7 (middle), and WT vs. DKO at day 10 (right). The blue and red lines indicate 0.5- and 2-fold levels of DKO (day 0), DKO (day 7), and DKO (day 10) relative to WT, respectively. (B) The circular diagram indicates the numbers of proteins, which were either upregulated or downregulated between two mouse groups. The number of animals is 3 each. (C) The results of GO analysis of the proteomic data show the numbers of downregulated proteins (written in blue) in the lungs of DKO mice compared with those in WT mice. The top 10 enrichments of GO biological processes (P < 0.05) for the downregulated proteins are listed (top), and the GO cellular components (P < 0.05) for the downregulated proteins are listed (bottom). GO terms related to cellular redox homeostasis and mitochondria are in blue, and others are in black.

### 3.4 Proteomic analysis revealed an increase in proteins related to adhesion and transport but a decrease in antioxidant enzymes in lung tissues of the DKO mice

To explore the mechanisms underlying metabolic remodeling, we performed proteomic analysis of lung tissues from DKO mice after Asc withdrawal (Supplementary Table 1). A volcano plot indicated that 36 proteins were upregulated and 23 proteins were downregulated in the Asc-supplemented DKO mice (day 0) compared with WT mice (Fig. 7A). Comparing the protein data of DKO mice (day 7) with WT mice, 28 proteins were increased and 28 proteins were decreased. Comparing the protein data of DKO mice (day 10) with those of WT mice, 38 proteins were increased and 24 proteins were decreased. There were 34 proteins that were not increased in DKO mice (day 0) but increased in DKO mice (day 7) or DKO mice (day 10), whereas there were 15 proteins that were not decreased in DKO mice (day 0) but decreased in DKO mice (day 7) or DKO mice (day 10) (Fig. 7B, bold). Characterization of these proteins based on the established GO knowledge base implied that the proteins increased in DKO mice were associated with cellular localization and component organization (Supplementary Table 4A). On the contrary, proteins decreased in DKO mice were associated with cellular redox homeostasis for “biological process” and mitochondria for “cellular component” (Fig. 7C), respectively. Redox-reactive proteins are highly susceptible to ROS and hence are prone to oxidative modification [40], which could promote their selective degradation.

### 3.5 Asc withdrawal caused protein oxidation and accelerated the subsequent degradation of oxidized proteins

**Fig. 8:**
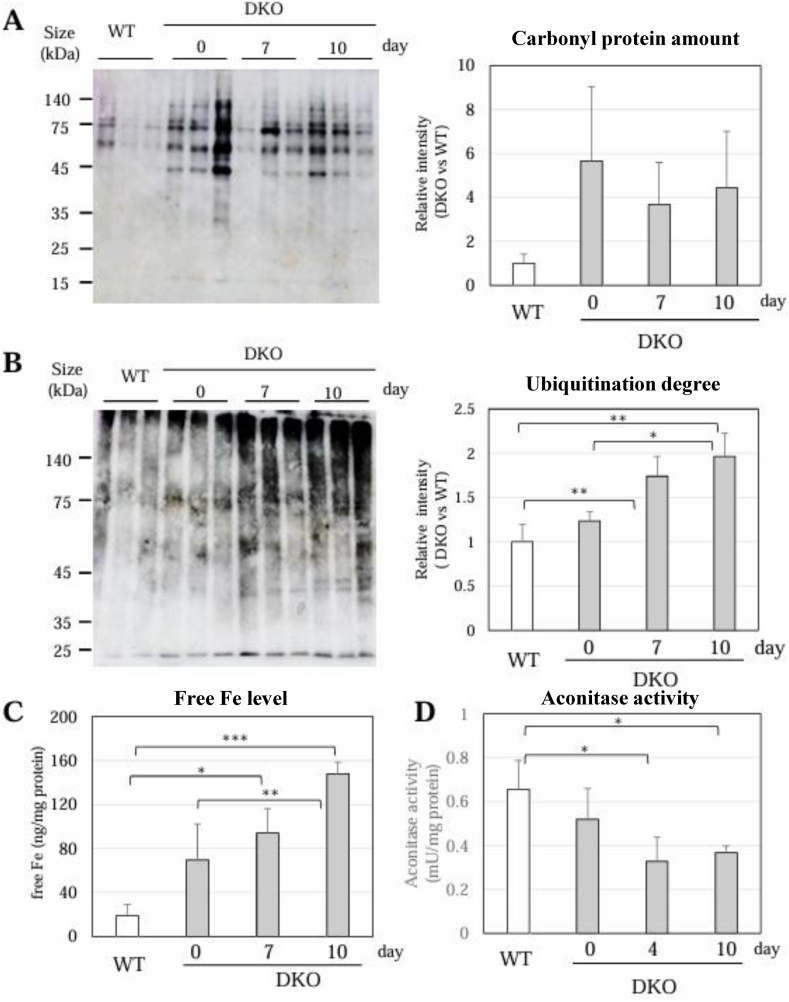
Changes in carbonyl proteins, polyubiquitinated proteins and free iron (A) Carbonyl proteins in lung homogenates were detected using a carbonyl protein assay kit. (B) Immunoblot analysis was performed on lung proteins using an antibody against ubiquitin. (C) Free iron in the lung tissue extracts was measured using an iron assay kit. (D) Aconitase activity was measured using an aconitase activity assay kit. Data are presented as the mean ± SE. The number of animals is 3 each. ∗, *P* < 0.05; ∗∗, *P* < 0.01; ∗∗∗, *P* < 0.001.

To evaluate the association of oxidation with these changes in metabolism and proteins, we performed analyses of oxidized proteins by detecting carbonylated proteins. The results indicated that carbonylated proteins were originally high in the lungs of the DKO mice even with Asc supplementation compared with those in the WT mice (Fig. 8A, Supplementary Table 5). Under SOD1 deficiency, Asc supplementation does not appear to sufficiently suppress oxidative damage in the lungs, where oxidative stress is stronger than in other organs. To our surprise, however, carbonylated proteins did not increase following Asc withdrawal even during the promotion of pulmonary damage. Since oxidized proteins are preferentially ubiquitinated and undergo selective degradation by 20S proteasome [41,42], we considered that Asc withdrawal stimulated the degradation of oxidized proteins via the ubiquitin-proteasome system. Immunoblot analysis using an antibody against ubiquitin actually showed an increased positivity rate (Fig. 8B). Thus, degradation of oxidized proteins could be promoted by the ubiquitin-proteasome system.

To examine whether oxidized proteins are actually degraded by the ubiquitin-proteasome system, we performed a similar analysis using the proteasome inhibitor MG132. When MG132 was administered intraperitoneally, ubiquitinated proteins accumulated, with little difference between the presence and absence of Asc (Supplementary Fig. 5A). Carbonyl proteins tended to be higher in DKO mice (day 7) than in Asc-supplemented DKO mice (day 0), but there was a large individual variability and no significant difference was observed (Supplementary Fig. 5B). We also compared metabolites in the lungs of DKO mice (day 0) and DKO mice (day 7) (Supplementary Table 6). When MG132 was administered intraperitoneally, amino acid levels tended to decrease compared with those of untreated mice, with little difference between DKO mice (day 0) and DKO mice (day 7). Furthermore, intraperitoneal administration of MG132 inhibited the thickening of the alveolar wall in DKO mice (day 7) (Supplementary Fig. 5C). These results collectively suggest that oxidized proteins are degraded by the ubiquitin-proteasome system, providing amino acids that are then used as materials for alveolar wall thickening.

Iron-sulfur (Fe–S) clusters in proteins are highly reactive to oxidative insult, as discussed in detail concerning iron-regulatory proteins, which is designated as cytosolic aconitase ACO1 here, and mitochondrial aconitase, designated as ACO2, upon exposure to ROS [43,44]. Moreover, a recent study has shown that Fe–S cluster-containing proteins in the electron transport chain of mitochondria are selectively damaged in the lungs of mice exposed to hyperoxia [45]. Because two crucial antioxidants were absent, we considered that iron metabolism was similarly impaired in the lungs of the DKO mice. As shown in Fig. 7C, free iron tended to be high in the DKO mice even with Asc supplementation and was strikingly elevated following Asc withdrawal. While Western blot analyses of proteins showed no changes in ACO1 and ACO2 proteins (Supplementary Fig. 5), the aconitase activity was actually decreased following Asc withdrawal (Fig. 8D). In the case of aconitase, the insertion of one Fe to 3Fe–4S is carried out reversibly in response to iron status, wherein the protein structure is preserved to a near-native state even after iron release [43,44], which could make iron-depleted ACO proteins resistant to proteolytic degradation.

## 4 Discussion

**Fig. 9:**
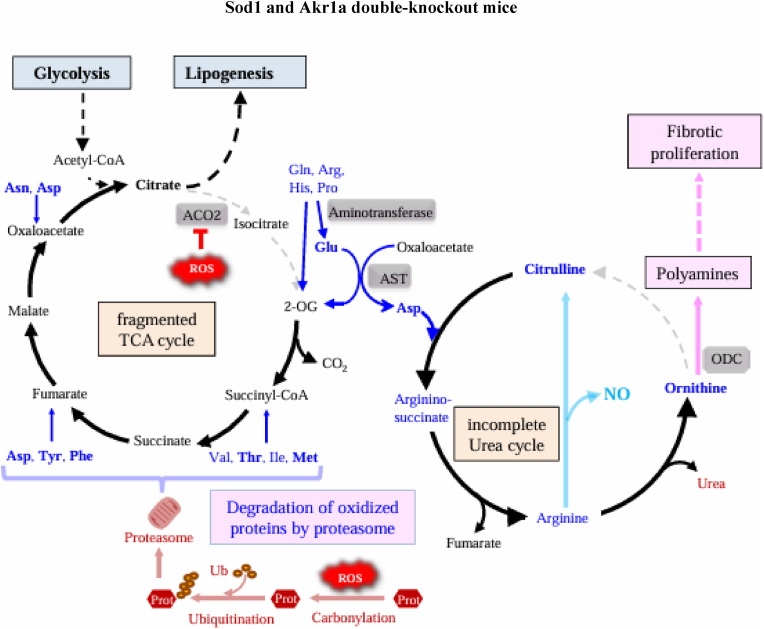
Schematic presentation of the process from oxidative protein modification to lung fibrosis Following Asc withdrawal, ROS elevate and fragment the TCA cycle in a process catalyzed by aconitase. In the meantime, oxidative modification of protein (Prot) stimulates polyubiquitination followed by proteasomal degradation, which releases amino acids. While carbon backbones of amino acids are catabolized in the fragmented TCA cycle, amino groups are transferred to Glu by corresponding aminotransferases followed by conversion to Asp by AST. Asp and citrulline are partially converted to ornithine in the incomplete urea cycle and eventually utilized to synthesize polyamines, which may stimulate fibrotic proliferation. Elevated amino acids in the lungs of DKO mice are shown in bold. Ub, ubiquitin.

DKO mice are able to survive for more than one year when supplemented with Asc. Upon Asc withdrawal, however, they experience persistent oxidative stress and die from pneumonia in a short period of time [24]. Employing this mouse model, we revealed the causal association by which oxidative injury promotes metabolic remodeling of lung parenchymal cells, which leads to the support of fibrotic proliferation. Amino acids, which are substrates for the remodeling of metabolic pathways, appeared to be supplied through selective degradation of oxidized proteins by the ubiquitin-proteasome system (Fig. 9). The resultant nitrogen from amino acid catabolism was, in part, recruited for polyamine synthesis through the incomplete urea cycle. Thus, it is conceivable that persistent ROS promoted protein oxidation followed by degradation, and released amino acids were metabolized in the remodeled pathways, which led to the support of fibrotic proliferation in the lung tissue (Fig. 9).

Analysis of metabolites indicated an accumulation of citrate/isocitrate, but also showed a decline in 2-OG content among intermediary compounds of the TCA cycle. Our nLC-MS/MS assay system does not distinguish diastereomers such as citrate and isocitrate. However, aconitase, which converts citrate to isocitrate, is the enzyme that is most sensitive to oxidative modification by ROS and reactive nitrogen oxide species (RNOS), including peroxynitrite [44]. We confirmed that the aconitase activity was decreased in the lung tissues following the Asc withdrawal (Fig. 8D). Since NOS2 expression was actually induced in the DKO mice, it is likely that not only ROS but also RNOS were involved in inhibition of the aconitase activity. Accordingly, it is conceivable that the impairment of aconitase activity caused citrate accumulation, which could have been recruited for fatty acid synthesis, but, instead, resulted in a decrease in the 2-OG levels. This type of metabolic change in the TCA cycle has previously been characterized in M1-polarized macrophages that dominantly act during pro-inflammation upon infectious stimuli [39]. Since cardiomyocytes in the homozygous knockout of Sod2 encoding mitochondrial manganese-containing SOD isoform also show this type of metabolic remodeling due to elevated ROS [46], both infiltrated macrophages and lung parenchymal cells in the DKO mice could experience the same metabolic remodeling in response to elevated ROS. The metabolic remodeling in the TCA cycle would allow more glucose-derived carbon compounds to flow into lipogenesis, based on biochemical interpretation and results on macrophages [39]. Since *de novo* synthesis of lipids, notably phospholipids, needs to be activated to promote the expanding cell size and membranous organelles in proliferating cells, the metabolic remodeling would be required to support fibrotic proliferation by supplying the building blocks of organelle membranes in the lung.

Cellular iron homeostasis is maintained by the iron-regulatory protein ACO1 via regulation of the translation of the mRNAs that are responsible for iron metabolism [43]. In the meantime, ACO2 catalyzes the conversion of citrate to iso-citrate in the TCA cycle but is also proposed to act as an iron-sensing regulator in mitochondria [47]. On the other hand, a recent study has shown that hyperoxia causes destabilization of the Fe–S cluster in proteins constituting the electron transport chain in mitochondria of mouse lungs [45]. Here GO analysis (Fig. 7C) and aconitase activity assay (Fig. 8D) show a decrease in mitochondrial proteins and function, leading us to hypothesize that ATP production *via* the TCA cycle followed by the respiratory chain was impaired. In addition to the defect in energy production by the impaired TCA cycle coupled with the electron transport chain, destruction of the Fe–S cluster releases free iron and hydrogen peroxide concomitantly, which could have collectively generated hydroxyl radicals, which is the most harmful type of ROS [44]. We actually observed an elevation of free iron in the lung tissue following the Asc withdrawal (Fig. 8C). Thus, in addition to the ROS produced by inflammatory cells, free iron elevated in the lungs is also a potent aggravating factor of alveolar damage. Involvement of necroptosis, which is a necrosome-mediated programmed cell death, has been proposed in inflammatory pulmonary injury [48]. Free iron plays a primary role in the execution of ferroptosis, an iron-dependent necrotic cell death, which is also involved in pulmonary diseases. However, our preliminary experiments did not yield convincing results regarding the type of cell death involved (data not shown), so we will leave this issue to future research.

It was surprising to see that oxidized proteins, as judged by the detection of carbonyl proteins, were originally higher in the DKO mice compared with that in the WT mice, but these were not significantly elevated following Asc withdrawal. Since proteins with polyubiquitination were elevated following Asc withdrawal, this seemingly contradictory phenomenon during the progressing tissue damage could be attributable to the accelerated degradation of oxidized proteins most likely via 20S proteasome [41,42]. This stimulated protein degradation could account for the source of amino acids that were catabolized in the remodeled TCA cycle, while oxidized proteins were removed.

Catabolic reaction of amino acid releases not only carbohydrates for energy metabolism but also nitrogen-containing compounds as a byproduct, which, if converted to ammonia, is toxic. Under physiological conditions, ammonia is largely transferred to the liver in the form of glutamine (Gln) after conjugation with glutamate (Glu) by means of glutamine synthetase, and is eventually metabolized to less-toxic urea through the urea cycle. Since glutamine synthetase is prone to oxidative inactivation [49], conjugation of ammonia with Glu may not proceed properly and could disturb nitrogen transfer to the liver in DKO mice. Although carbamoyl phosphate synthetase I and ornithine transcarbamylase, which are essential enzymes for the detoxification of ammonia in the urea cycle, are not expressed in organs other than the liver or intestine, residual enzymes constituting the urea cycle are still expressed [50,51]. Metabolite analyses of lung tissues revealed increases in the content of citrulline and ornithine, which are amino acids unique to the urea cycle. Arginase, which hydrolyzes arginine (Arg) to urea and ornithine, is particularly induced by inflammatory stimuli [52]. Since nitrite levels were very low (less than 0.6 nmol/mg protein), arginase-mediated hydrolysis of Arg to urea and ornithine likely exceeded NOS2-mediated conversion to NO and citrulline. These results collectively suggest that amino acid-derived nitrogen is, at least in part, metabolized through the following processes, instead of producing ammonia. The amino group of some amino acids are first transferred to 2-OG to form Glu by means of aminotransferase specific to the donor amino acid, followed by transferal to oxaloacetate by means of aspartate aminotransferase (AST), which results in the production of aspartate (Asp) [53]. The coupling of Asp and citrulline generates arginosuccinate, which is in turn converted to Arg by the release of fumarate. While fumarate is recruited to the TCA cycle, Arg is hydrolyzed to urea and ornithine by arginase. These reactions can be accomplished even in an incomplete urea cycle, but lack of ornithine transcarbamylase disables the conversion of ornithine to citrulline. Under this situation, ornithine can be recruited to the branched pathway for polyamine synthesis, which is initiated by ornithine decarboxylase (ODC) [54]. Despite no significant changes in the polyamine content *per se* in our metabolite analysis, marked accumulation of the polyamine metabolite *N*-acetylspermidine is evidence of the actual synthesis of polyamines. Because enzymes for nucleic acid synthesis, which is another nitrogen-utilizing pathway, were downregulated, more nitrogen could be recruited for synthesizing polyamines under this situation. While polyamines are potentially protective against inflammatory injury, they are also well-known promoters of cell growth [55,56]. On the other hand, enzymatic oxidation of polyamines produces amino aldehydes, which are themselves cytotoxic, and deamination by polyamine oxidase produces acrolein, which is an aldehyde that is highly toxic to cells [57]. Therefore, the resultant polyamines could aggravate fibrotic tissue remodeling by inducing parenchymal cell damage followed by a promotion of the proliferation of fibrotic cells. Nitric oxide synthase activity is induced upon inflammatory stimuli and produces citrulline from Arg along with the production of NO [58]. This type of urea cycle-related metabolic remodeling accompanies oxidative insult and reportedly associates with the pathogenesis of a variety of pulmonary diseases such as cystic fibrosis, asthma, and chronic obstructive pulmonary disease (COPD) [2]. For example, cystic fibrosis is a disease that exhibits impaired mucociliary clearance, which increases the risk of death from respiratory failure. In cystic fibrosis patients, amino acids characteristically increase, and both ROS production and polyamine synthesis are also stimulated [59]. Considering the similarities in these issues, the metabolic remodeling revealed by the current study could also occur in such patients and could even be involved in the pathogenesis of these respiratory diseases. We consider the metabolic remodeling to primarily be an adaptive response to inflammation in the lungs.

## 5 Conclusions

Based on analyses of the DKO mice, we propose that the following reactions eventually led to death by respiratory failure. Withdrawal of Asc under SOD1 deficiency markedly elevates ROS levels that promote the oxidative modification of susceptible proteins — notably a group of Fe–S-containing proteins. Oxidized proteins are prone to polyubiquitination and selective degradation by proteasome, which releases massive amounts of amino acids. While one nitrogen in urea comes from Arg, the other comes from Asp. Asp receives amino group from Glu through the action of AST, and Glu receives the amino group from many other amino acids through the action of the corresponding aminotransferases. Taken these reactions together, Asp is thought to play an intermediary role in supplying nitrogen from amino acids used for catabolism to the urea cycle. While the resultant polyamines have anti-inflammatory effects, they also promote the fibrotic proliferation of cells. These collective events lead to pulmonary tissue remodeling under oxidative stress, which results in respiratory failure and death.

## CRediT authorship contribution statement

**Tsukasa Osaki:** Formal analysis, Funding acquisition, Investigation. **Takujiro Homma:** Investigation. **Yuya Soma:** Investigation. **Satoshi Miyata:** Resources. **Yumi Matsuda:** Supervision. **Junichi Fujii:** Conceptualization, Funding acquisition, Project administration, Supervision, Validation, Writing – original draft.

## Data availability

The raw data and analysis files have been deposited to the ProteomeXchange Consortium *via* the jPOST partner repository [60] with the data set identifier PXD049470 (JPST002941).

## Funding information

This work was supported in part by a JSPS KAKENHI Grant-in-Aid for Scientific Research (C) [grant number 23K06410] to TO and [grant number 21K06850] to JF from the Japan Society for the Promotion and Science (JSPS) and the YU-COE program [grant number S6] to and JF from Yamagata University.

## Declaration of competing interest

We declare no conflicts of interest between the authors or with any institution in relation to the content of this article.
